# Herbal medicine Sihogayonggolmoryeo-tang or Chai-Hu-Jia-Long-Gu-Mu-Li-Tang for the treatment of post-stroke depression: A protocol for a systematic review and meta-analysis

**DOI:** 10.1097/MD.0000000000012384

**Published:** 2018-09-21

**Authors:** Chan-Young Kwon, Boram Lee, Sun-Yong Chung, Jong Woo Kim, Aesook Shin, Ye-yong Choi, Younghee Yun, Jungtae Leem

**Affiliations:** a Chung-Yeon Medical Institute, Gwangju; b Department of Clinical Korean Medicine, Graduate School, Kyung Hee University; c Department of Korean Medicine, Kyung Hee University Korean Medicine Hospital at Gangdong; d Research and Development Institute, CY Pharma Co.; e Dongshin Korean Medicine Hospital, Seoul, Republic of Korea.

**Keywords:** depression, east asia traditional medicine, protocol, review, stroke

## Abstract

**Introduction:**

This systematic review protocol describes the methods that will be used to evaluate the efficacy and safety of herbal medicine Sihogayonggolmoryeo-tang (SGYMT) or Chai-Hu-Jia-Long-Gu-Mu-Li-Tang for the treatment of post-stroke depression.

**Methods and analysis:**

The following electronic databases will be searched up to July 2018 without language or publication status restrictions: MEDLINE, the Cochrane Central Register of Controlled Trials (CENTRAL), EMBASE, Allied and Complementary Medicine Database (AMED), Cumulative Index to Nursing, and Allied Health Literature (CINAHL), and PsycARTICLES. We will also search Korean and Chinese databases. Any clinical randomized controlled trials related to SGYMT treatment for post-stroke depression will be included. Changes in the degree of depression and adverse events will be assessed as primary outcomes. The total effective rate and changes in neurological function, activities of daily living, and quality of life will be evaluated as secondary outcomes. Study selection, data extraction, assessment of study quality, and evaluation of the quality of evidence for the main findings will be performed independently by 2 researchers. The data synthesis and analysis will be performed using RevMan version 5.3. The results will be expressed as a risk ratio for dichotomous data and as the mean difference or standardized mean difference for continuous data. Data will be synthesized by either a fixed-effects or random-effects model according to a heterogeneity test or the number of studies included in the meta-analysis. The methodological quality of the included studies will be evaluated using the Cochrane Collaboration's risk of bias tool. The quality of evidence for each main outcome will be evaluated using the Grading of Recommendations Assessment, Development, and Evaluation (GRADE) approach.

**Ethics and dissemination:**

Ethical approval is not required because individual patient data are not included. The findings of this systematic review will be disseminated through a peer-reviewed publication or conference presentations.

**PROSPERO registration number:**

CRD42018102939

## 1 Introduction

Stroke is a major cause of disability and mortality worldwide.^[1]^ Post-stroke depression (PSD) is one of the most common neuropsychiatric complications that occurs after a stroke and has been studied actively since the 1980s.^[2]^ In the Diagnostic and Statistical Manual of Mental Disorders, Fifth Edition (DSM-5), PSD is categorized as a depressive disorder due to another medical condition, which is divided into disorders with depressive features, a major depressive-like episode, or mixed features.^[3]^ As PSD is generally regarded as major depressive-like episodes that occur after a stroke, this disorder can be defined as having at least 5 symptoms of major depression in the 2 weeks after a stroke.^[4]^

Recent epidemiological studies have shown that PSD occurs in 30% to 35% of patients with stroke.^[5–7]^ Although there is a report that PSD was not significantly different from depression in other populations,^[8]^ this disease is important clinically because it can have substantial impacts on rehabilitation, health recovery, compliance, disability, quality of life, and even stroke recurrence and mortality in patients with stroke.^[9–11]^

Although the underlying neurobiological mechanism of this disease is still poorly understood, psychosocial factors such as distress caused by the disability and poor social support and biological factors such as amine neurotransmitter dysregulation and proinflammatory cytokine release are described as the main etiologies.^[12,13]^ Moreover, in a recent meta-analysis, a history of mental illness, the female sex, old age, neuroticism, family history of mental disorders, stroke severity, and degree of disability were found to be risk factors for PSD.^[14]^

Early treatment of PSD can improve physical and cognitive recovery in patients with stroke and can increase survival rates.^[2]^ Generally, antidepressants are recommended for the treatment of PSD;^[15]^ however, there are side effects such as increased risks of fracture, stroke, and mortality.^[16,17]^ Importantly, 2 recently published cohort studies noted that the use of antidepressants may increase the risk of stroke recurrence.^[18,19]^ Antidepressants also are associated with a risk of a manic episode during PSD treatment.^[20]^ Therefore, there is a need for safe and effective alternatives to conventional pharmacotherapy for PSD.

As one of those alternatives, herbal medicine (HM) derived from traditional East Asian medicine, including traditional Chinese medicine (TCM), traditional Korean medicine (TKM), and Kampo medicine, can be considered. For thousands of years, HM, which is a combination of medicinal herbs, has been used to treat and prevent various diseases. A recent network meta-analysis revealed that HM may have a significant beneficial effect on recovery after stroke.^[21]^ In Chinese clinical practice guidelines (CPGs), HM has been introduced for the treatment or prevention of PSD.^[20]^

Sihogayonggolmoryeo-tang (SGYMT) is composed of 11 herbs including Bupleuri Radix, Pinelliae Rhizoma, Cinnamomi Ramulus, Poria, Scutellariae Radix, Jujubae Fructus, Ginseng Radix or Codonopsis Radix, Ostreae Concha, Fossilia Ossis Mastodi, Zingiberis Rhizoma Recens, and Rhei Rhizoma, and was first introduced in the classical Chinese text “Treatise on Cold Damage Diseases.” Since then, the decoction has been used clinically for the treatment of psychological anxiety, insomnia, neurosis, and irritability. Moreover, in the TKM CPGs for depression, SGYMT was recommended recently for the treatment of major depressive disorder (MDD) and PSD.^[22]^ In a rat model of depression induced by chronic stress exposure, SGYMT showed significant antidepressant effects by preventing dysfunction in the prefrontal cortex.^[23]^ Additionally, in a study conducted in a mouse model of despair, long-term administration of SGYMT also was reported to have an antidepressant effect such as that of imipramine, a tricyclic antidepressant.^[24]^ Apart from these antidepressant effects, the administration of SGYMT for 3 months was reported to significantly increase high-density lipoprotein cholesterol (HDL-C) in patients with mild-to-moderate hypertension in a randomized controlled trial (RCT) in Japan.^[25]^ This suggests that SGYMT may inhibit stroke recurrence indirectly by controlling dyslipidemia, a risk factor for ischemic stroke.

As can be seen, SGYMT is a promising candidate for PSD intervention; however, there has been no systematic review regarding SGYMT in the treatment of PSD. Therefore, in this review, we will investigate the current evidence on the efficacy and safety of SGYMT in PSD.

## 2 Methods and analysis

The protocol for this systematic review has been registered in the International Prospective Register of Systematic Reviews (PROSPERO) (registration number, CRD42018102939) on July 26, 2018. We will conduct a systematic review according to this protocol, but if protocol amendments occur, the dates, changes, and rationales for each amendment will be tracked in PROSPERO. This protocol is reported in accordance with the Preferred Reporting Items for Systematic Review and Meta-Analysis Protocols (PRISMA-P) 2015 statement^[26]^ and the Cochrane Handbook for Systematic Reviews of Interventions.^[27]^

### 2.1 Data sources and search strategy

The following databases will be searched comprehensively from their inception to July 2018 by 2 independent researchers (C-YK and BL): 6 English-language databases (MEDLINE via PubMed, EMBASE via Elsevier, the Cochrane Central Register of Controlled Trials [CENTRAL], the Allied and Complementary Medicine Database [AMED] via EBSCO, the Cumulative Index to Nursing and Allied Health Literature [CINAHL] via EBSCO, and PsycARTICLES via ProQuest), 5 Korean-language databases (Oriental Medicine Advanced Searching Integrated System, Koreanstudies Information Service System [KISS], Research Information Service System [RISS], Korean Medical Database [KMbase], and Korea Citation Index [KCI]), and 2 Chinese-language databases (China National Knowledge Infrastructure [CNKI], and Wanfang Data). We will also search the reference lists of the relevant articles and perform a manual search on Google Scholar to identify additional trials. We will include not only the literature published in journals but also “gray literature” such as theses and conference proc0eedings. There will be no language restriction.

The search terms will be composed of the disease term part (e.g., “[depression or depressive] and stroke”) and the intervention term part (e.g., “Chai-Hu-Jia-Long-Gu-Mu-Li-Tang or Chai-Hu-Jia-Long-Gu-Mu-Li-Wan or Chai-Hu-Jia-Long-Gu-Mu-Li-Pian or Simo granule or Saikokaryukotsuboreitou or Saikokaryukotsuboreito or Sihogayonggolmoryeo-tang”). The search strategies for the MEDLINE and EMBASE are shown in Table 1 and will be modified and used similarly for the other databases.

**Table 1:**
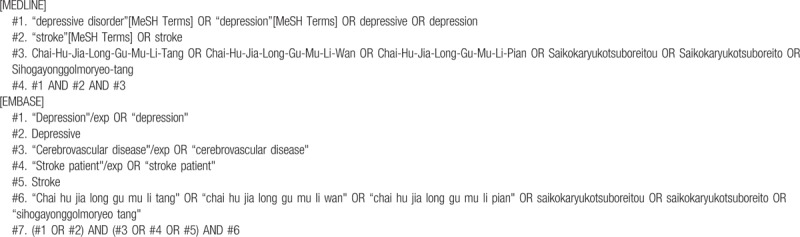
Search strategies for the MEDLINE and EMBASE.

### 2.2 Inclusion criteria

#### 2.2.1 Types of studies

Only RCTs will be included. Studies using inappropriate random sequence generation methods such as alternate allocation or allocation by birthdate will be excluded. If only the expression “randomization” is mentioned without the randomization methods, it will be regarded as an RCT and will be included in this review. However, the risk of bias of the related item will be evaluated as high. We will include both parallel and crossover studies. In crossover studies, only first-phase data will be used to calculate the effect size and in the meta-analysis. Other designs such as in vivo, in vitro, case reports, and retrospective studies will be excluded.

#### 2.2.2 Types of participants

We will include studies on patients with a diagnosis of depression following stroke using standardized diagnostic tools such as the DSM. There will be no restriction on the sex, age, or race of the participants. Studies will be excluded if the participants have drug allergies or other serious illnesses such as cancer, liver disease, or kidney disease.

#### 2.2.3 Types of interventions

Studies on SGYMT as experimental interventions will be included. We will also include studies on modified SGYMT, which contains >50% of the original prescription composition. Studies involving SGYMT combined with other therapies as experimental interventions will be included if the other therapies are used equally in both the experimental and control groups. The use of any form of SGYMT will be allowed. Studies comparing different types of HM will be excluded. There are no other restrictions regarding the control intervention.

#### 2.2.4 Types of outcome measures

The primary outcome measures are as follows:

1. Change in the degree of depression as measured by validated assessment tools such as the Hamilton Depression Scale^[28]^ and Beck Depression Inventory.^[29]^
2. Adverse events as measured by the Treatment Emergent Symptom Scale^[30]^ or the incidence.

The secondary outcome measures are as follows:

1. Total effective rate: The total effective rate is a nonvalidated outcome measure that is processed secondarily according to certain evaluation criteria such as clinical symptom improvement or the improvement rates of other quantified outcomes. In the assessment of the total effective rate, participants are generally classified as “cured,” “markedly improved,” “improved,” or “non-responder” after treatment. The total effective rate is calculated consistently using the following formula: *Total effective rate = N1* *+* *N2* *+* *N3 / N*, wherein *N1*, *N2*, *N3*, and *N* are the number of patients who are cured, markedly improved, improved, and who comprise the sample size, respectively.
2. Changes in neurological function as measured by validated assessment tools such as the National Institutes of Health Stroke Scale^[31]^ and the Scandinavian Stroke Scale.^[32]^
3. Changes in activities of daily living measured by validated assessment tools such as the Barthel Index,^[33]^ the Functional Independence Measure (FIM),^[34]^ and the Stroke Impact Scale.^[35]^
4. Quality of life as measured by validated assessment tools such as the Stroke Specific Quality of Life Scale^[36]^ and the 36-Item Short Form Health Survey.^[37]^

### 2.3 Study selection

**Figure 1:**
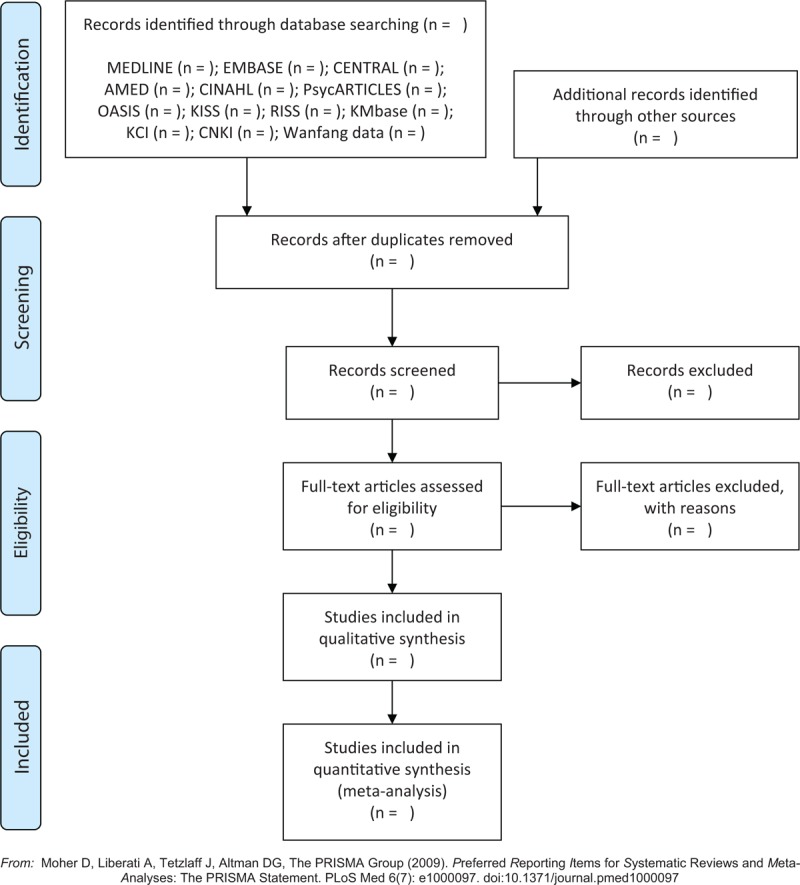
A PRISMA flow diagram of the literature screening and selection processes. AMED = Allied and Complementary Medicine Database, CENTRAL = Cochrane Central Register of Controlled Trials, CINAHL = Cumulative Index to Nursing and Allied Health Literature, CNKI = China National Knowledge Infrastructure, KCI = Korea Citation Index, KISS = Koreanstudies Information Service System, KMbase = Korean Medical Database, OASIS = Oriental Medicine Advanced Searching Integrated System, RISS = Research Information Service System.

The study selection will be conducted by 2 independent researchers (C-YK and BL) according to the above selection criteria. After removing duplicates, we will evaluate the titles and abstracts of the searched studies for relevance and then evaluate the full texts of the remaining studies for eligibility. Any disagreement on study selection will be resolved through discussion with other researchers. Quotations from included articles will be made available online to researchers using Zotero (Roy Rosenzweig Center for History and New Media at George Mason University, Fairfax, VA), a reference management software program. The literature selection process will be reported in accordance with the PRISMA guidelines^[38]^ (Fig. 1).

### 2.4 Data extraction

Using a standardized data collection form, 2 independent researchers (C-YK and BL) will perform and crosscheck the data extraction. Discrepancies will be resolved through discussion with other researchers.

The extracted items will include the first author's name; year of publication; country; sample size and number of dropouts; details about the participants, intervention, and comparisons; duration of the intervention; outcome measures; and adverse events associated with SGYMT. In particular, regarding the data on interventions, the name, source, dosage form, and dosage of each medical substance as well as the principles, rationale, and interpretation of the intervention form will be extracted by consulting the Consolidated Standards of Reporting Trials Extension for Chinese Herbal Medicine Formulas 2017.^[39]^

The extracted data will be recorded using Excel 2007 (Microsoft, Redmond, WA) and will be shared among researchers using Dropbox (Dropbox, Inc., CA) folders. We will contact the corresponding authors of the included studies via e-mail to request additional information if the data are insufficient or ambiguous.

### 2.5 Quality assessment

Two independent researchers (C-YK and BL) will assess the methodological quality of the included studies and the quality of evidence for each main finding. Discrepancies will be resolved through discussion with other researchers.

The methodological quality of the included studies will be assessed using the Cochrane Collaboration's risk of bias tool.^[40]^ We will assess random sequence generation, allocation concealment, blinding of participants and personnel, blinding of outcome assessments, incomplete outcome data, selective reporting, and other biases for each included study. Each domain will be categorized into 1 of 3 groups: “low risk,” “unclear,” or “high risk.” Each evaluation will be recorded in an Excel 2007 (Microsoft) spreadsheet and will be shared among researchers in Dropbox (Dropbox, Inc) folders. The evaluated results will be presented in a full review using Review Manager version 5.3 software (Cochrane, London, UK).

The quality of evidence for each main outcome will be evaluated by using the Grading of Recommendations Assessment, Development, and Evaluation (GRADE) approach.^[41]^ According to the GRADE standards, we will assess the risk of bias; inconsistency, indirectness, and imprecision of the results; and the probability of publication bias using a four-item scale (“very low,” “low,” “moderate,” or “high”). The results of the quality of evidence will be presented through a Summary of Findings table. The evaluation process will be shared among researchers using the online program GRADEpro (https://gradepro.org/).

### 2.6 Data synthesis and analysis

The data synthesis and analysis will be performed using Review Manager version 5.3 software (Cochrane) and will be shared among researchers in Dropbox (Dropbox, Inc) folders. Descriptive analyses of the details of the participants, interventions, and outcomes will be conducted for all included studies. A quantitative synthesis will be performed if there are studies using the same types of intervention, comparison, and outcome measure. The data will be pooled as the mean difference or standardized mean difference with 95% confidence intervals (CIs) for continuous outcomes and as a risk ratio with 95% CIs for dichotomous outcomes. Heterogeneity between the studies in terms of effect measures will be assessed using both the chi-squared test and the *I*^2^ statistic. We will consider *I*^2^ values >50% and 75% indicative of substantial and high heterogeneity, respectively. In the meta-analyses, a random-effects model will be used when the heterogeneity is significant (*I*^2^ >75%), whereas a fixed-effects model will be used when the heterogeneity is nonsignificant. A fixed-effects model will be also used when the number of studies included in the meta-analysis is very small, wherein inter-study variance estimates have poor accuracy.^[42]^ When it is considered that the heterogeneity is too high for the results to be synthesized (*I*^2^ >75%), a subgroup analysis will be conducted as follows to determine the cause of heterogeneity.

### 2.7 Subgroup analysis

If heterogeneity is evaluated as significant (*I*^2^ >75%) and the necessary data are available, we will conduct a subgroup analysis to account for the heterogeneity. A subgroup analysis will be conducted according to the following criteria: the treatment period; the dosage forms of SGYMT, such as decoctions, granules, and so on; the presence or absence of a placebo; and the severity of depression.

### 2.8 Sensitivity analysis

To identify the robustness of the meta-analysis result, we will perform sensitivity analyses by determining the effects of excluding studies with high risks of bias, studies with missing data, and outliers.

### 2.9 Assessment of reporting bias

If there are >10 trials included in the analysis, reporting biases such as publication bias will be assessed by using funnel plots. When reporting bias is implied by funnel plot asymmetry, we will attempt to explain possible reasons.

## 3 Ethics and dissemination

Ethical approval will not be needed because the data used in this systematic review will not be individual patient data and there will be no concerns regarding privacy. The results will be disseminated by the publication of a manuscript in a peer-reviewed journal or presentation at a relevant conference.

## 4 Discussion

Although PSD is the most common psychiatric complication of stroke, existing therapies have limitations in terms of their efficacy and 0safety.^[16,17]^ HM, and specifically SGYMT, which is a promising candidate for the treatment of depressive disorders, may be considered effective alternatives or complementary therapies to conventional therapies for PSD. Relevant clinical studies and animal studies have been conducted to date; however, the evidence was not evaluated systematically. Therefore, this study will examine the efficacy and safety of SGYMT in treating PSD systematically.

SGYMT has been used traditionally for psychological anxiety, insomnia, neurosis, or irritability, and recently has been suggested as a therapeutic option for the treatment of MDD and PSD in TKM CPGs.^[22]^ In addition, as HM is a therapeutic approach with multiple targets and various effects can be expected,^[43]^ the clinical effects may affect not only depressive symptoms but also outcomes such as health status, rehabilitation, neurological symptoms, and quality of life in patients with PSD. Thus, in this review, we will analyze the effect of SGYMT on the depressive symptoms of patients with PSD as the primary outcome and its effects on a wide variety of symptoms including neurological function, activities of daily living, and quality of life as secondary outcomes. We will also review the potential side effects of SGYMT and herb–drug interactions through a safety analysis.

We believe that the results of this systematic review will help clinicians optimize treatment protocols for patients with PSD and will introduce safe and effective PSD treatment strategies for use in clinical settings. In addition, the results of this review will also provide patients with PSD a broader range of evidence-based HM options. Policy makers will be able to identify the basis for HM as a candidate to reduce the disease burden of PSD in public health settings. Finally, researchers will be able to broaden the applications of HM in their research by identifying evidence that HM has multiple targets rather than a single target.

## Author contributions

**Conceptualization:** Chan-Young Kwon.

**Methodology:** Chan-Young Kwon, Boram Lee, Jungtae Leem.

**Writing – original draft:** Chan-Young Kwon, Boram Lee.

**Writing – review & editing:** Sun-Yong Chung, Jong Woo Kim, Aesook Shin, Ye-yong Choi, Younghee Yun, Jungtae Leem.

**Supervision:** Jungtae Leem.
